# Expression of several *Phytophthora cinnamomi* putative *RxLR*s provides evidence for virulence roles in avocado

**DOI:** 10.1371/journal.pone.0254645

**Published:** 2021-07-14

**Authors:** Melissa Joubert, Robert Backer, Juanita Engelbrecht, Noëlani van den Berg

**Affiliations:** 1 Forestry and Agricultural Biotechnology Institute, University of Pretoria, Pretoria, Gauteng, South Africa; 2 Department of Biochemistry, Genetics and Microbiology, University of Pretoria, Pretoria, Gauteng, South Africa; Osmania University, INDIA

## Abstract

*Phytophthora cinnamomi* is a plant pathogenic oomycete that causes Phytophthora root rot of avocado (PRR). Currently, there is a limited understanding of the molecular interactions underlying this disease. Other *Phytophthora* species employ an arsenal of effector proteins to manipulate host physiology, of which the RxLR effectors contribute to virulence by interfering with host immune responses. The aim of this study was to identify candidate RxLR effectors in *P*. *cinnamomi* that play a role in establishing PRR, and to infer possible functions for these effectors. We identified 61 candidate *RxLR* genes which were expressed during infection of a susceptible avocado rootstock. Several of these genes were present in multiple copies in the *P*. *cinnamomi* genome, suggesting that they may contribute to pathogen fitness. Phylogenetic analysis of the manually predicted RxLR protein sequences revealed 12 *P*. *cinnamomi* RxLRs that were related to characterised effectors in other *Phytophthora* spp., providing clues to their functions *in planta*. Expression profiles of nine more *RxLR*s point to possible virulence roles in avocado–highlighting a way forward for studies of this interaction. This study represents the first investigation of the expression of *P*. *cinnamomi RxLR* genes during the course of avocado infection, and puts forward a pipeline to pinpoint effector genes with potential as virulence determinants, providing a foundation for the future functional characterization of RxLRs that contribute to *P*. *cinnamomi* virulence in avocado.

## Introduction

*Phytophthora cinnamomi* Rands is a soil-borne, hemibiotrophic, plant-pathogenic oomycete. It is globally distributed and affects at least 5000 plant species, including economically important crops such as avocado, macadamia, peach and chestnut. *P*. *cinnamomi* typically infects the roots of host plants causing root rot, as well as stem cankers and dieback of shoots [1]. The wide host range of the pathogen has led to devastating impacts on biodiversity in natural ecosystems in Europe and Australia, where *P*. *cinnamomi* infection poses a major threat to forests and natural flora [2–5]. In the agricultural industry, the oomycete causes Phytophthora root rot (PRR) of avocado, which has had a devastating impact on this crop [6–9]. Control of PRR is primarily through the application of chemicals such as phosphites, the use of good agricultural practices, and the planting of tolerant or resistant rootstocks [1,10]. Research into mechanisms of resistance against the pathogen is essential, since eradication is unlikely once it has established in soil [11].

Once a plant has been infected with *P*. *cinnamomi*, disease symptoms appear as a result of the release of effector proteins by the pathogen. These effector molecules are used by *Phytophthora* spp. to manipulate host plants [12,13]. According to the broadly accepted models of plant-pathogen interactions, effector proteins of most phytopathogens are important in eliciting specific responses in the host plant, by acting either as virulence or avirulence effectors [14]. Oomycetes use two different classes of effectors to contribute to pathogen virulence. Apoplastic effectors are secreted into the cell apoplast and function outside of host cells, whereas cytoplasmic effectors are secreted directly into the host cells in which they function [15].

One group of cytoplasmic effectors in *Phytophthora* spp. have a common R-x-L-R amino acid motif in their N-terminal regions, which has been hypothesised to play a role in their translocation and localisation inside host cells [16–18]. The conserved RxLR motif has enabled the identification of hundreds of putative RxLR effectors in various *Phytophthora* genomes [19–22], though the functional characterisation of the identified effectors remains an ongoing process.

In *P*. *cinnamomi*, several candidate *RxLR* genes have been predicted, none of which have been functionally characterised to date. Reitmann *et al*. [23] described the expression of 44 putative *RxLR* genes in cysts and germinating cysts of *P*. *cinnamomi* produced *in vitro*. McGowan & Fitzpatrick [24] identified 68 candidate *P*. *cinnamomi* RxLR effectors, using a combination of several prediction methods. A study by Hardham & Blackman [1] predicted 171 candidate RxLRs in the *P*. *cinnamomi* genome, based on similarity to candidate RxLRs in *Phytophthora infestans*. Most recently, 181 putative *RxLR* genes have been predicted in a new genomic analysis of *P*. *cinnamomi*, and 41 of these had altered expression profiles 5 days after inoculation of avocado [25]. This is the only study conducted to date which highlights which predicted RxLRs may have virulence roles in avocado, though the use of a single late timepoint in that study means that many questions remain unanswered regarding which *P*. *cinnamomi* RxLR effectors may specifically contribute to virulence during the establishing of PRR in avocado.

Unfortunately, functional characterisation of the putative RxLR effectors of *P*. *cinnamomi* has proven to be a challenge, especially since this oomycete is particularly recalcitrant to transformation [26]. With transformation of *P*. *cinnamomi* being so difficult to establish, common methods of protein functional characterisation—such as induction of gene overexpression, or generation of knockout mutants–cannot be used to infer the function of RxLR effectors in this pathogen. It is therefore necessary to establish alternative ways to narrow down candidates for functional assays in order to ultimately determine virulence roles of these pathogen effectors.

This study aimed to identify candidate RxLR effectors in *P*. *cinnamomi* that play a role in establishment of PRR in avocado, by focusing on the discovery of putative *RxLR* genes which were upregulated during infection of a susceptible avocado rootstock. The expression profiles of potential *RxLR* genes in *P*. *cinnamomi* were investigated using dual RNA-sequencing data. The candidate genes were manually annotated and their resultant protein sequences were predicted. Finally, these protein sequences were investigated for evolutionary relatedness to other *Phytophthora* effectors to assign putative functions.

## Results

### *RxLR* effector genes in *P*. *cinnamomi* are expressed during avocado infection

A library of putative *P*. *cinnamomi RxLR* effector genes was constructed using available sequence lists of candidates predicted in recent literature [1,23–25] as well as a set of 192 candidate *RxLR*s predicted in this study using a bioinformatics pipeline adapted from Win *et al*. [22]. Gene names for the full list of candidates were determined using a BLAST search of the recently available *P*. *cinnamomi* genome [25], and redundant sequences were removed. The final compiled library contained 322 candidate *RxLR* sequences from the *P*. *cinnamomi* GKB4 genome. This list of 322 candidates was further narrowed down to a final set of 238 candidate *RxLR*s (S1 Table) which were confirmed to have signal peptides according to SignalP 5.0 [27].

To narrow down the set of 238 candidate effector genes to those that were likely to play a role in infection of avocado, data generated from a dual RNA-Seq experiment were used. The reads generated from the RNA-Seq experiment were aligned to the *P*. *cinnamomi* genome and quantified. Normalised expression outputs from DESeq2 were extracted using the gene identities (IDs) from the GKB4 genome of the 238 candidate *RxLR*s to determine which were expressed *in planta* at 12 hours-, 24 hours- and 120 hours post-inoculation (hpi), when normalised against their expression in mycelia. In this study, the 12- and 24 hpi timepoints were taken to represent the biotrophic stage of infection (roots harvested were still healthy) [28] whereas the 120 hpi timepoint was representative of the necrotrophic growth stage (visually observed as total necrosis of the root tissue).

**Fig 1:**
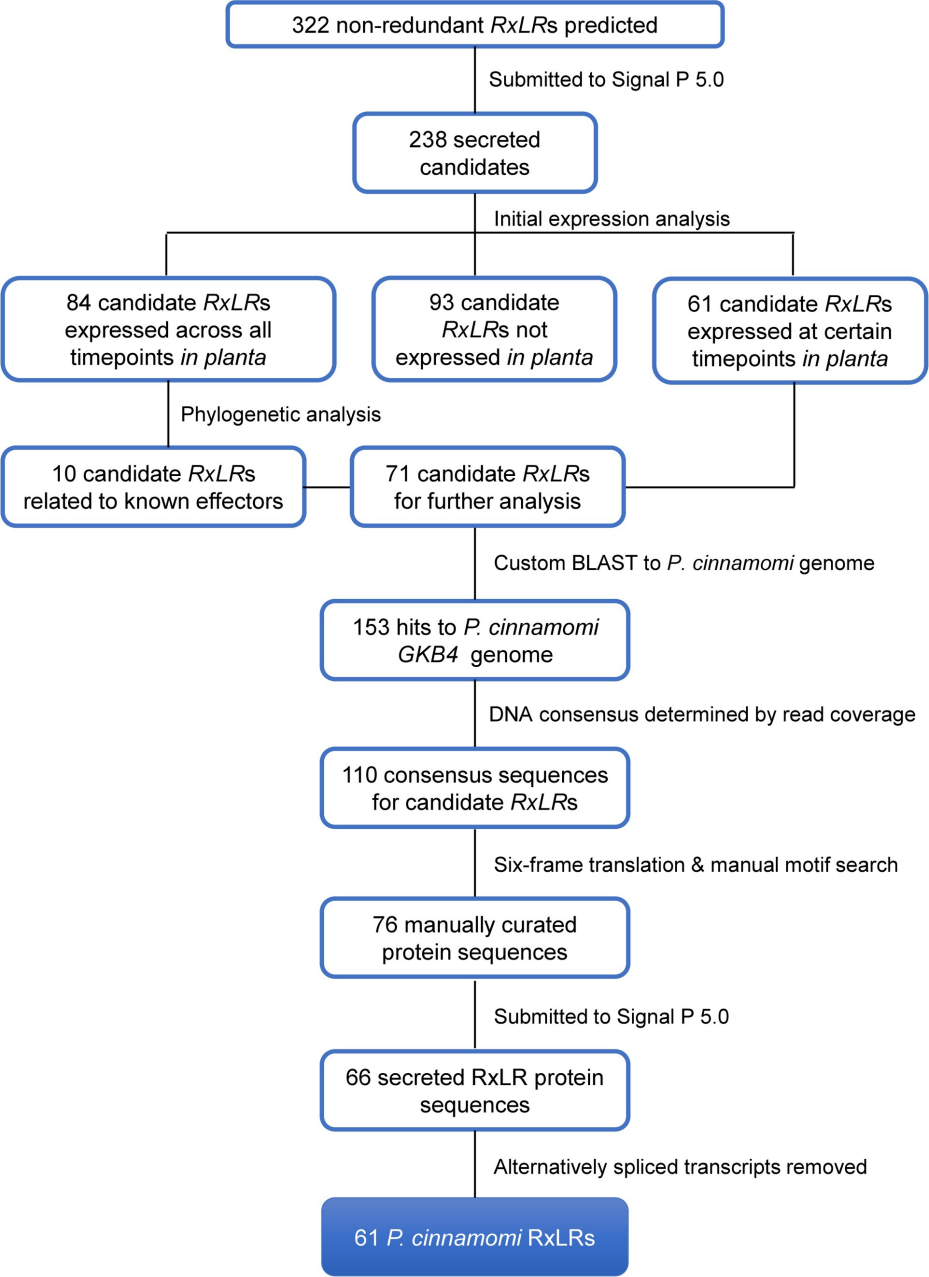
Number of *Phytophthora cinnamomi* RxLR effectors at different stages of the prediction pipeline. The number of RxLRs inferred at each step are shown in blue blocks. The workflow is indicated by black text next to black lines in the diagram. This pipeline was used to analyse the original list of 322 candidate *RxLR*s using the GKB4 *P*. *cinnamomi* genome to filter out candidates which may not contribute to virulence in avocado. The outcome of this process was the manual prediction of 61 protein sequences for *P*. *cinnamomi* RxLRs.

According to a preliminary screen of RNA-Seq data, 145 of the 238 candidate *RxLR* genes were expressed during infection of the susceptible avocado rootstock, when read counts at the different timepoints were compared to read counts in *P*. *cinnamomi* mycelia. Candidate genes which were upregulated at either the biotrophic or necrotrophic stage of infection (61 candidate genes) were selected for further analysis (S2 Table). The remaining 84 of the 145 expressed candidates were upregulated across all time points relative to the control. The expression profiles of these 84 genes did not meet selection criteria in this analysis, since functional effector genes are expected to be upregulated specifically at either early or late infection time points, but not both. The candidates expressed at all timepoints were thus screened for similarity to characterised RxLR effectors in other species, before 74 of the 84 candidates were discarded. Preliminary phylogenetic analyses resulted in the remaining 10 candidates being retained along with the 61 candidates which originally met the expression criteria. A total of 71 candidate *RxLR*s were thus retained for further analyses (Fig 1).

### Candidate *RxLRs* are present as paralogs in the *P*. *cinnamomi* genome

Genomic coordinates were obtained for each of the 71 candidate *RxLRs* which were kept based on their expression in avocado (S3 Table). The majority of the sequences (46 out of 71) had multiple hits to the *P*. *cinnamomi* genome. All possible genomic locations were recorded for each of the 153 hits to the genome (S3 Table), and consensus sequences were generated for each of the candidate genes based on the coverage of each respective genomic region by *P*. *cinnamomi* transcripts from the dual RNA-Seq experiment. DNA consensus sequences were successfully generated for a total of 110 predicted genomic locations of the 71 candidate *RxLR*s (Fig 1). Positions of introns were manually recorded for each candidate gene, and alternatively spliced transcripts were noted (S4 Table).

Eight genomic loci did not have genes already predicted for the genome by BRAKER2; these were manually annotated and designated *mg00001*-*mg00008* in the GKB4 genome (shown in S1 Table). Four candidate genes, *PcinRxLR52a*, *PcinRxLR52b*, *PcinRxLR68b* and *PcinRxLR68c*, had incorrectly predicted coding regions; these were re-annotated using the correct genomic coordinates (listed in S4 Table).

### A number of prospective paralogs do not produce RxLR effector proteins

Once the 110 consensus sequences had been generated for the coding regions (CDSs) of candidate *RxLRs*, they were translated into six reading frames to predict the correct protein sequence for each putative effector. Thirty-four translated candidate genes did not contain an RxLR motif downstream of a Methionine (M) residue in any of the reading frames; these genes were considered to be falsely predicted candidate *RxLRs*. Final protein sequences could be predicted for the remaining 76 annotated CDSs of candidate *RxLR*s, which included two splice variants each for *PcinRxLR31b*, *PcinRxLR68c* and *PcinRxLR69a*, and three splice variants for *PcinRxLR31c*. Ten of the manual protein predictions that produced full-length proteins did not contain a signal peptide and were discounted as true RxLRs. A total of 66 peptide sequences were thus manually predicted (S4 Table) for 61 genomic loci containing putative *RxLR* genes (Fig 1).

### Phylogenetic analysis reveals evolutionary relatedness with characterised RxLRs from other *Phytophthora* spp.

After the protein sequences of the 61 remaining candidate RxLRs had been determined following manual annotation, redundant *P*. *cinnamomi* protein sequences (where multiple gene copies had the same predicted protein sequences) were removed prior to phylogenetic analysis. This resulted in 42 unique *P*. *cinnamomi* candidates being subjected to phylogenetic analyses to investigate whether the informed protein sequences could reveal possible functions for the candidate *P*. *cinnamomi* RxLR effectors based on their evolutionary relatedness to characterised effectors of other species.

**Fig 2:**
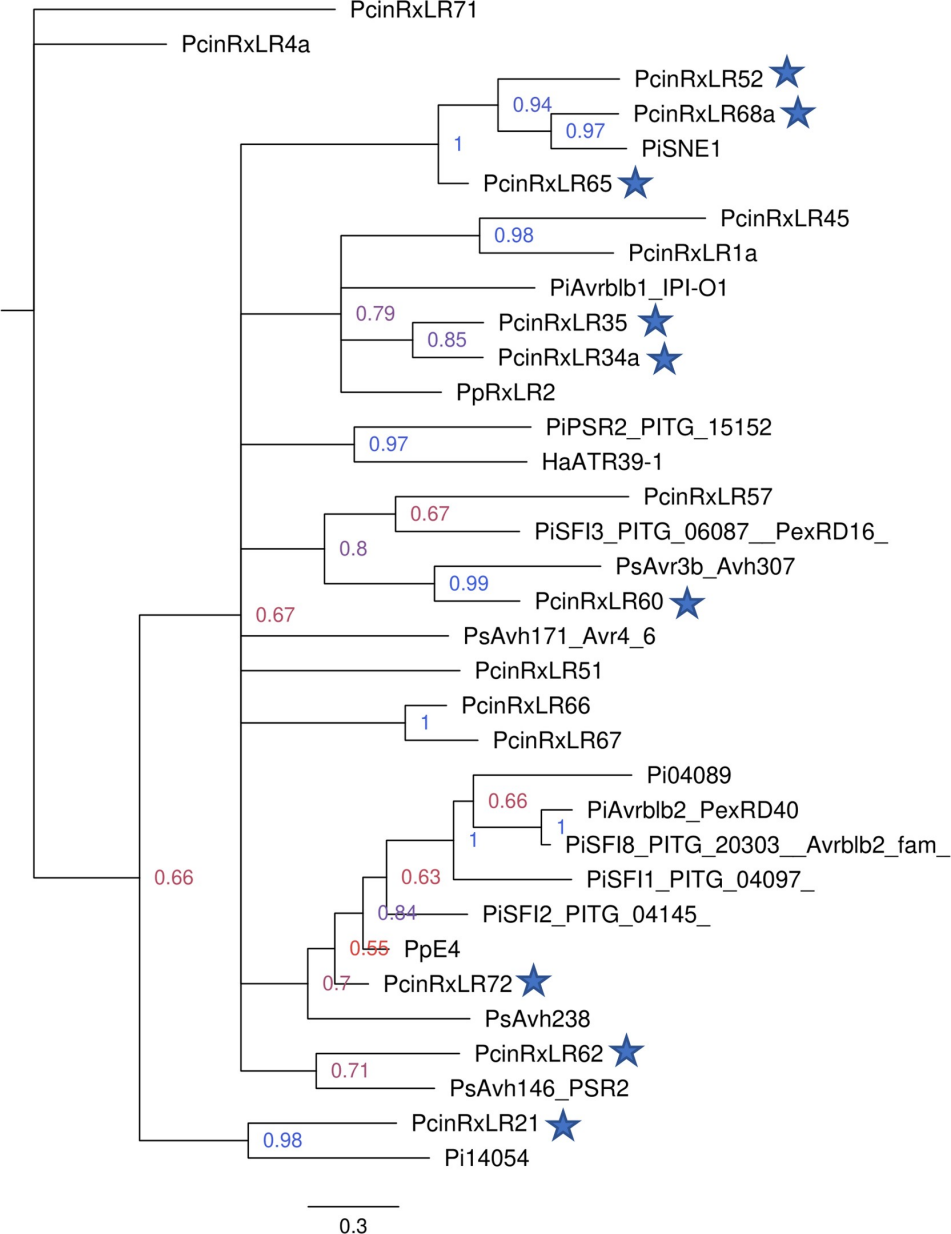
Evolutionary relatedness of *Phytophthora cinnamomi* candidate RxLRs to characterised effector proteins from other oomycete species. A phylogenetic tree was produced from Bayesian inference analysis of the predicted N-terminal regions of the *P*. *cinnamomi* candidate RxLR effectors aligned with the N-terminal regions of functionally characterised RxLRs in other species. Uninformative sequences from a preliminary phylogenetic analysis were removed to produce the refined tree above. Support for branches is indicated by posterior probability values, shown up to the second significant digit for each node. Probability value colours fall on a spectrum ranging from red (lowest probability values) to blue (highest probability values). Proteins indicated by blue stars are candidate *P*. *cinnamomi* RxLR effectors which grouped with characterised RxLRs from other species with a posterior probability equal to or higher than 0.7.

The phylogenetic tree resulting from Bayesian inference analysis contained several sequences without similarity to any other sequence, which formed their own outgroups (S1 Fig). No functional inferences could thus be made for these 25 candidate *P*. *cinnamomi* effectors since they were not shown to be evolutionarily related to any of the characterised RxLR effectors from other species included in this study. These sequences were removed from the dataset used for the second phylogenetic analyses to increase the resolution of clades that did show similarity of candidate *P*. *cinnamomi* RxLRs with known effectors. The remaining sequences were aligned and a second Bayesian inference analysis was performed. The phylogenetic tree with increased resolution of clades is shown in Fig 2.

The refined tree, like the original, contained several smaller clades of two to eight protein sequences each, though there was no significant statistical support for relatedness between these small clades. Grouping within the clades revealed that some of the *P*. *cinnamomi* candidate effectors exhibited similarity to characterised RxLRs from other species, allowing functional inferences to be made for those candidates. Nine *P*. *cinnamomi* RxLRs which grouped with characterised effectors with a probability above 0.7 are indicated by blue stars in Fig 2.

The candidate *P*. *cinnamomi* effectors PcinRxLR52, PcinRxLR68a and PcinRxLR65 formed a clade with *P*. *infestans* PiSNE1, with a posterior probability of more than 0.9 to support their evolutionary relatedness. PcinRxLR60 grouped with *P*. *sojae* Avr3b with a high statistical support value of 0.99. A third *P*. *cinnamomi* candidate, PcinRxLR21, grouped with a known effector, *P*. *infestans* Pi14054, with high support (0.98), forming its own outgroup.

Candidates that were more distantly related to known effectors were PcinRxLR62, which grouped with *P*. *sojae* Avh146 (probability of 0.71), and PcinRxLR57, which grouped with *P*. *infestans* PiSFI3 (probability of 0.67). PcinRxLR34a and PcinRxLR35 were both shown to share evolutionary history with *P*. *infestans* Avrblb1 and *P*. *parasitica* RxLR 2, with a supported probability of 0.79. Another candidate with more distant evolutionary inferences was PcinRxLR72, which showed divergence from *P*. *sojae* Avh238 (probability of 0.7) and, more recently, from *P*. *parasitica* PpE4, though this inference had a much lower support value of 0.55.

For the most part, this phylogenetic analysis confirmed evolutionary groupings shown in the original tree (S1 Fig), though *P*. *cinnamomi* candidates PcinRxLR4a and PcinRxLR71 formed a separate outgroup in the final tree. Interestingly, PcinRxLR51 and *P*. *sojae* Avh171, which grouped together with low statistical support in the original tree (probability of 0.65), did not form clades with each other or any other RxLRs in the second alignment; highlighting the value of repeating the phylogenetic analysis in this pipeline.

### Alignment of related sequences confirms similarity to characterised effectors

To investigate whether the phylogenetic inferences made from N-terminal alignments of proteins were relevant when the full-length peptides were analysed, alignments were generated for the proteins which grouped within the smaller clades the final phylogenetic tree using their complete amino acid sequences. The full alignments are shown in S2 Fig.

Full alignments for sequences with the highest relatedness to known effectors, with probability values higher than 0.9, confirmed the probability of shared evolutionary history. In the alignment of *P*. *cinnamomi* candidates PcinRxLR52a and PcinRxLR68a with *P*. *infestans* PiSNE1, several regions within the first 130 residues were conserved across all three peptides (S2A Fig), while even more residues were shown to be conserved between only PcinRxLR68a and PiSNE1, providing supporting evidence for their common evolutionary pathway.

The alignment of PcinRxLR21 and Pi14054 showed little overall similarity between the two peptides, though several single residues were conserved across the length of the peptides (S2B Fig), suggesting a more distant relatedness than was inferred from the phylogenetic tree. In contrast, the alignment of PcinRxLR60 with *P*. *sojae* Avr3b revealed large numbers of conserved residues across the entire length of the sequences, separated by short regions of dissimilar residues (S2C Fig), confirming the likelihood that these effector genes diverged from a single ancestral gene. The alignment of PcinRxLR72 with *P*. *sojae* Avh238 also had greater overall similarity, with several short regions of consensus separated by short regions of divergent residues (S2D Fig), providing support for their divergence from an ancestral effector.

When PcinRxLR34a and PcinRxLR35 were aligned with *P*. *infestans* Avrblb1 and *P*. *parasitica* RxLR 2, low overall similarity was visible in the consensus (not shown), but when this alignment was refined to exclude *P*. *infestans* Avrblb1, more similarity was observed, though most of the conserved residues were observed in the N-terminal regions of the peptides (S2E Fig). Finally, in the alignments of PcinRxLR62 with *P*. *sojae* Avh146 (S2F Fig) and PcinRxLR57 with *P*. *infestans* PiSFI3 (S2G Fig), several short regions of conserved residues were separated by longer spans of dissimilar residues. This suggests a more distant evolutionary relatedness for these effectors, and so functional inferences were not made for these highly divergent sequences.

### Several putative *P*. *cinnamomi RxLR* genes are upregulated at 12-, 24- and 120 hpi *in planta*

Once phylogenetic analyses were complete, the expression profiles for selected *P*. *cinnamomi* candidate *RxLR*s were re-examined in more detail using an alternative software to estimate transcript abundance. The chosen genes included all candidates which had shown evolutionary relatedness to characterised RxLRs from other oomycetes as well as candidates which showed interesting expression peaks at one or more timepoints during avocado infection in our initial screen of upregulated *RxLR*s. For the repeated analysis, fold change of 61 candidate genes was again compared to expression in mycelia, but gene identities used to mine RNA-Seq data included those which were manually annotated in this study. Interestingly, multiple copies of candidate effectors did not share identical expression profiles, and so expression of each copy of *P*. *cinnamomi* candidate *RxLR*s was determined individually. Fold change of all 61 genes for the time-course analysis is shown in S5 Table.

**Fig 3:**
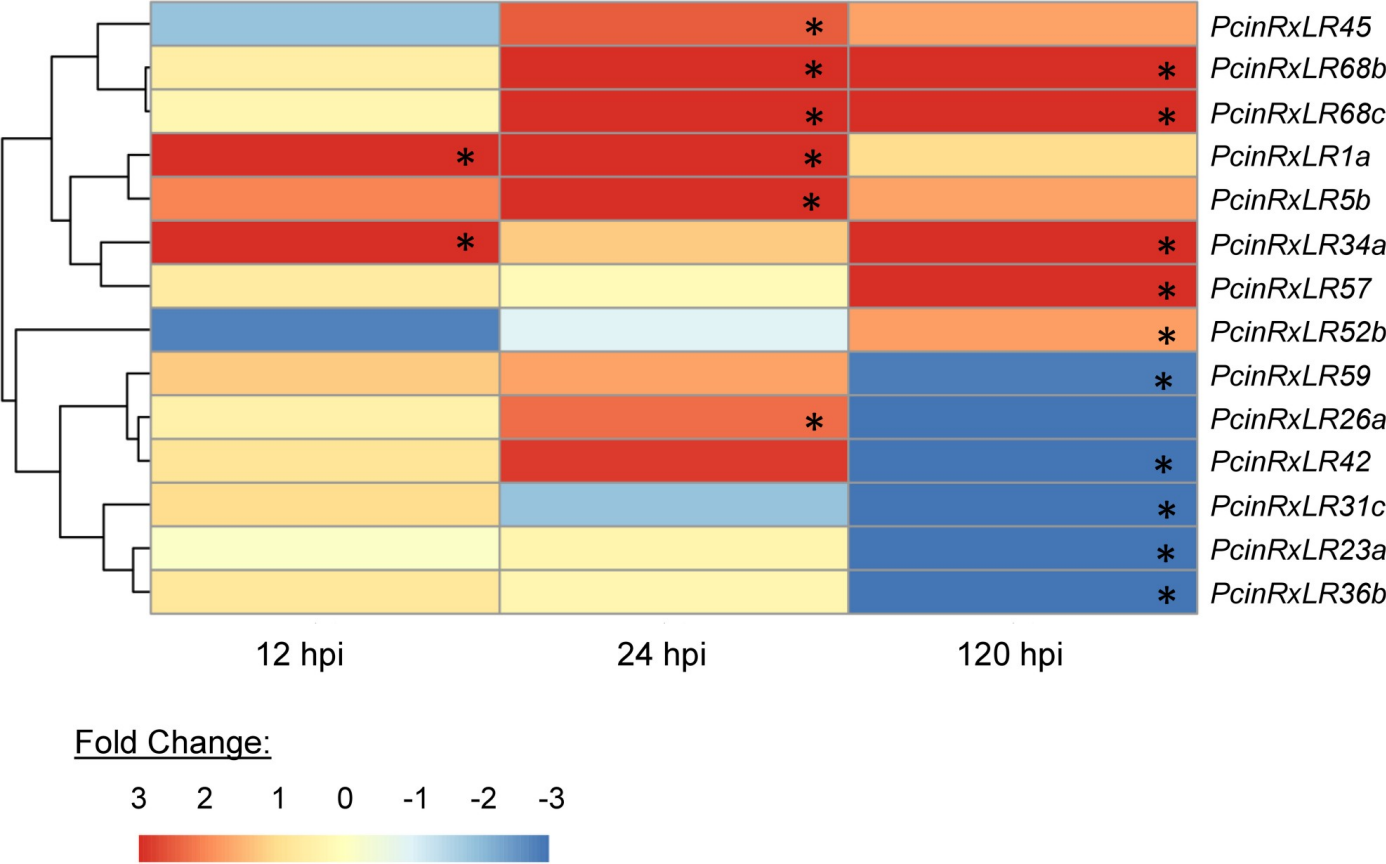
Expression profiles of 14 significantly expressed *Phytophthora cinnamomi* candidate *RxLR*s in infected avocado. Gene expression was visually represented in a heatmap based on read counts generated from dual RNA-Seq data. Expression levels are indicated as fold change of each gene normalized against expression in mycelia, and colour-coded according to the corresponding scale. Expression profiles were generated using RNA-Seq data obtained for three biological replicates of a susceptible avocado rootstock, R0.12, harvested at 12 hours-, 24 hours- and 120 hours post inoculation (hpi). Statistical significance was determined according to DESeq2 outputs, and is indicated on colour blocks for fold change relative to mycelia at relevant timepoints for each gene. *Significant expression at time-point relative to expression in mycelia at p ≤ 0.05.

Based on their expression peaks at different timepoints, 33 candidates were discarded from further analysis. These genes were either up- or down-regulated at all timepoints during infection of avocado, or there was no change in their expression *in planta* when compared to mycelia. The expression of the remaining 28 candidates was visualised in a heatmap (S3 Fig) and statistical significance of expression peaks was evaluated. Further investigation showed that 14 *P*. *cinnamomi RxLR*s were significantly up- or down-regulated at one or more timepoints during the course of infection (p ≤ 0.05), as shown in the heatmap in Fig 3.

Eight of the candidate *P*. *cinnamomi RxLR*s were more highly expressed during the biotrophic stages of infection, represented by the 12 hpi and 24 hpi timepoints, when compared to the *in vitro* sample used as a control. Three candidates, *PcinRxLR1a*, *PcinRxLR5b* and *PcinRxLR26a* were significantly upregulated during early timepoints. The remaining candidate genes, *PcinRxLR23a*, *PcinRxLR31c*, *PcinRxLR36b*, *PcinRxLR42* and *PcinRxLR59* were all significantly downregulated during the necrotrophic stage of *P*. *cinnamomi* infection (represented by the 120 hpi timepoint) when compared to their expression in mycelia. By contrast, the candidate genes *PcinRxLR57* and *PcinRxLR52b* were significantly upregulated at 120 hpi, suggesting roles in establishing necrotrophy. The four remaining candidates, *PcinRxLR34a*, *PcinRxLR45*, *PcinRxLR68b* and *PcinRxLR68c*, all had significantly increased expression at both 24 hpi and 120 hpi, relative to their expression *in vitro*, but not at 12 hpi,. The expression of these putative effectors at both of these timepoints warrants further investigation to determine whether they play a role during biotrophic or necrotrophic growth stages of the pathogen.

### *P*. *cinnamomi RxLR*s expressed *in planta* are similar to uncharacterised candidate RxLR effectors from other species

To determine whether the 28 candidate genes with expression patterns of interest were similar to putative RxLR effectors from other oomycete species, the *P*. *cinnamomi* RxLR protein sequences were subjected to a BLASTp search of the NCBI database. Results of the search (shown in S6 Table) indicated that candidate effector PcinRxLR1a did not exhibit similarity to predicted proteins from any other species. Four putative effectors, PcinRxLR21, PcinRxLR30, PcinRxLR42 and PcinRxLR59 were similar (57.7–73.7%) to uncharacterised avirulence homolog proteins, Avh448, Avh114, Avh141 and Avh320, respectively, in *P*. *sojae* [29]. PcinRxLR4a, PcinRxLR4b and PcinRxLR7 had top hits to other potential RxLR proteins in *P*. *sojae*, all designated as “avirulence protein 1b” due to their similarity to characterised *P*. *sojae* effector Avr1b-1 [29]. PcinRxLR45 was 90% identical to a serine protease from *P*. *sojae*.

The 19 remaining *P*. *cinnamomi* candidate RxLR effectors exhibited similarity to hypothetical proteins and candidate RxLR effector proteins in various *Phytophthora* species, with identity scores ranging from 46.9% to 87.0% (S6 Table). None of the candidate effectors had notable hits to RxLR effectors with known function, though this may change in future analyses as the functions of other RxLR effectors are elucidated by researchers in this field.

## Discussion

Several genomes of *Phytophthora* species contain hundreds of candidate RxLR effectors [20–22,29], and many of these effectors have been demonstrated by functional studies to contribute to virulence during host infection. In *P*. *cinnamomi*, recent studies have identified candidate *RxLR*s in the pathogen’s genome [1,23–25] but it remains unclear whether these putative effector genes are expressed during plant infection to contribute to virulence, and functions have yet to be assigned to any *P*. *cinnamomi* effectors. To that end, this study identified 61 candidate *P*. *cinnamomi RxLR* effector genes which are specifically expressed during the initial infection of avocado. We also used a novel approach to investigate whether putative virulence functions could be assigned to these effectors based on similarity to characterised RxLRs in other *Phytophthora* species.

In this study, a compilation of predicted candidate RxLRs in *P*. *cinnamomi* was created using data produced in previous studies [1,23–25] as well as the set of 192 candidate *RxLR*s we identified in the *P*. *cinnamomi* genome using the pipeline developed by Win *et al*. [22]. The final dataset consisted of 238 putative *P*. *cinnamomi* RxLRs with confirmed signal peptides. While this library constitutes the largest number of predicted RxLRs for *P*. *cinnamomi* to date, the number of *RxLR* effectors predicted in this study is likely an underestimate of the true number of *P*. *cinnamomi RxLR* candidates, since several prediction methods used require the presence of an exact RxLR motif in the N-terminal regions of the sequences [22]. Other researchers have shown that variants to the traditional RxLR motif exist [30–33]. It should also be noted that the number of *P*. *cinnamomi RxLR*s in our initial library were restricted to those with BRAKER2 annotations in the GKB4 genome, and so it is possible that a set of putative effectors could have been excluded as a result of the incomplete annotation of the genome. Manual curation of the entire *P*. *cinnamomi* genome using available RNA-Seq data would eliminate this artefact from future genetic studies.

Of the 238 candidate *RxLR*s predicted in the genome, only 61 were predicted to play a role during *P*. *cinnamomi* infection of avocado based on their expression profiles *in planta*. This is comparable to observations made in *P*. *sojae*, in which only a small subset of the predicted RxLR effectors appear to contribute to virulence in the soybean host [34]. It remains unclear, however, whether the subset of *P*. *cinnamomi* effectors expressed in our dataset represent a set of key effectors which contribute to virulence in all hosts, or whether the set of expressed *RxLR*s would differ depending on the host plant that is infected, or the time-points evaluated in any given study.

Based on their expression profiles and initial phylogenetic analyses, 71 putative *RxLR*s were retained for further investigation and several of these genes had multiple hits to different contigs of the *P*. *cinnamomi* genome [25]. In most cases, the hits differed slightly in their overall similarity scores, leading to the hypothesis that the hits represented duplications of the candidate *RxLR*s, rather than errors in the genome assembly.

When these genomic coordinates were investigated for coverage by RNA-Seq reads, several of the proposed duplications were not expressed. Even though they were similar to regions which were expressed, hits to genomic regions without corresponding transcriptome evidence were not used to generate final protein sequences. It is possible that the non-expressed duplications were located in regions of the genome that were not transcriptionally active at the timepoints used in this study, or were not expressed during the infection of the avocado host specifically.

The presence of several candidate *RxLR*s in multiple copies in the *P*. *cinnamomi* genome reflect their roles as putative effectors. Several *RxLR* effector genes are present as multiple copies in the genome of *P*. *sojae*, the copy numbers of which can vary between different strains of the pathogen [35,36]. It has been hypothesised that the presence of some *RxLR* effector genes as multiple copies may contribute to the fitness of the pathogen [36].

The final protein sequences of RxLR effectors were produced based on manual annotation. These sequences were used in a phylogenetic analysis to investigate whether functional inferences could be made for the RxLRs during infection of avocado, based on shared evolutionary history between RxLR effectors. Putative functions could be assigned to six of the candidate effectors based on their consistent grouping with characterised effectors across all phylogenetics analyses. PcinRxLR34a and PcinRxLR35 showed relatedness to *P*. *parasitica* RxLR 2 in both trees, and an alignment of these protein sequences revealed short regions of consensus between the peptides. It is therefore possible that PcinRxLR34a and PcinRxLR35 may have similar functions to *P*. *parasitica* RxLR 2, which suppresses programmed cell death (PCD) in host cells [37]. This hypothesis is supported by the expression of *PcinRxLR35* which peaks during early infection timepoints (S5 Table), though this upregulation was not statistically significant. Interestingly, *PcinRxLR34a* was shown to be significantly upregulated at 12 hpi and 120 hpi (Fig 3), and has previously been shown to be expressed (as *Phyci_30885*) in pre-infection structures of the pathogen [23]. The expression of this gene during the necrotrophic stage of infection is contrary to a function in suppressing cell death, but its anomalous expression profile is similar to that of *P*. *parasitica* RxLR 2, which was expressed at both early and late timepoints in a susceptible host, *Citrus sunki* [37]. The contrary expression peaks of this gene thus provides an interesting avenue for further investigation.

In both the first and second phylogenetic analysis, PcinRxLR21 was shown to be related to *P*. *infestans* Pi14054, though support for this inference differed between the two trees. Alignment of the full peptide sequences confirmed several conserved residues between the two proteins, but separation of these conserved residues by longer spans of dissimilar amino acids suggests that these effectors are more divergent than inferred by the 0.98 supporting probability in the second tree. Nonetheless, distant relatedness could still mean that PcinRxLR21 functions similarly to Pi14054, which suppresses RNA silencing in host plants during biotrophic infection [38]. This suggestion is tentatively supported by the upregulation of *PcinRxLR21* at 12 hpi and 24 hpi and its downregulation during necrotrophic infection at 120 hpi, though these expression peaks were not statistically significant in this study.

Another clade with high supporting probability values was the group containing PcinRxLR68a, PcinRxLR52, PcinRxLR65 and *P*. *infestans* PiSNE1, and further evidence for the relatedness between these peptides was provided by the alignment of full-length protein sequences. This is supported by observations made in literature, where Reitmann *et al*. [23] proposed *PcinRxLR65* (*Phyci_16230*) as a putative ortholog of *PiSNE1*, and Hardham & Blackman [1] predicted *PcinRxLR68a* (*Phyci_24296*) and *PcinRxLR68b* (*Phyci_297058*) as candidate *P*. *cinnamomi* RxLRs based on their homology to *PiSNE1*. It can therefore be postulated that all three of these candidates function to suppress necrosis during the biotrophic phase of host infection [39]. This hypothesis is supported by the expression profile of *PcinRxLR52a*, which peaks at 12 hpi and 24hpi, though this upregulation was not significant. In contrast to this postulation, both *PcinRxLR68b* and *PcinRxLR68c* were significantly upregulated at 24 hpi and 120 hpi (Fig 3), and *PcinRxLR52b* expression was suppressed at 12 hpi but increased at 120 hpi (Fig 3). Expression of these genes during the necrotrophic growth of the pathogen contradicts a potential function in suppression of necrosis, and is contrary to the expression of *PiSNE1* at early infection timepoints [39,40]; thus the function of these candidate effectors warrants further investigation in future studies. PcinRxLR68a and PcinRxLR65 were not significantly upregulated during infection of avocado at the time points sequenced in this study, though it was previously shown that *PcinRxLR65* is expressed in cysts and germinating cysts of *P*. *cinnamomi* [23]. It is therefore possible that these genes were expressed at earlier timepoints of infection that were not analysed in this study, or that these putative effectors function in virulent interactions with other host species. Future studies should include a wider range and larger number of infection timepoints in avocado, and compare expression of the genes in alternate host species, in order to provide better insight into uncharacteristic expression profiles of pathogen effector genes investigated in this study.

The opposing expression patterns of the multiple isoforms of many of the candidate genes in this study are an interesting observation, since expression polymorphisms have been found in the *RxLR*s of other *Phytophthora* species [36]. The differing expression patterns of gene isoforms in literature have only been shown for the same gene found in different isolates of a species [36]. To our knowledge, there have been no expression polymorphisms observed to date for multiple copies of the same gene within a single *Phytophthora* isolate.

One possibility is that *RxLRs* with multiple copies but distinct expression profiles represent recent duplications, in which case the effector genes are in the process of undergoing subfunctionalisation or neofunctionalisation–where one or more of the copies is in the process of diverging functionally from its paralogs [41]. This type of functional divergence due to differing expression profiles has been observed for multi-copy genes in several plant species, though the expression divergence of these genes refers to the specific tissues in which they are expressed, rather than the timing of their expression [42–44]. Although there have not yet been studies into this type of expression divergence for multi-copy genes in oomycetes, it remains a possibility that multi-copy *RxLRs* in this study represent paralogs that are in the process of evolving to carry out differing functions during infection by *P*. *cinnamomi*.

In phylogenetic analyses of the candidate RxLRs in this study, the majority of predicted *PcinRxLR*s which had expression profiles resembling those of effectors identified in other species did not show similarity to any functionally characterised RxLRs. This is understandable, since only a fraction of the candidate RxLRs identified in other species have been assigned functions to date, and so it is conceivable that the related effectors in other species have not yet been characterised. A BLAST search of the NCBI database using *P*. *cinnamomi* RxLR protein sequences revealed that the majority of the candidates were similar to uncharacterised hypothetical proteins and putative RxLR effectors in other *Phytophthora* species. Though none of the candidates were notably similar to functionally characterised RxLR effectors in other species, functional inferences for these *P*. *cinnamomi* putative RxLRs may be possible in the future, as functions of more candidate RxLR effectors in other *Phytophthora* species are revealed in years to come. It should also be noted that PcinRxLR45 was highly similar to a *P*. *sojae* housekeeping protein, which may indicate a fused-gene misannotation of this candidate in the *P*. *cinnamomi* GKB4 genome. The downregulation of this gene at 12hpi and significant upregulation at 24hpi supports its annotation as a candidate RxLR effector rather than a housekeeping protein, however. Future studies and molecular characterisation will reveal whether this putative *RxLR* gene was misannotated by the genome assembly, or whether the candidate RxLR contributes to virulence using functions resembling the serine protease.

In this study, *PcinRxLR1a*, *PcinRxLR5b* and *PcinRxLR26a* were all significantly upregulated during the biotrophic phase of infection, as is the norm for most known RxLR effectors [20,34,39,45,46], but they were not related to any known RxLRs in other species. These putative effectors, along with several others which were significantly downregulated during the necrotrophic phase of infection, remain promising candidates for RxLR effectors which contribute to *P*. *cinnamomi* virulence. A set of *P*. *sojae RxLR*s have been shown to be expressed during later infection stages [47], and so *P*. *cinnamomi* candidates with later expression peaks were not excluded from this study. Their presence in multiple copies in the genome further supports the conclusion that they are pathogen effectors likely to play a role during avocado infection.

Several *P*. *cinnamomi* candidate RxLRs were proposed to be related to RxLRs with known functions in other *Phytophthora* species, but functional similarity was not supported by expression of these effectors *in planta*. Despite this contradiction in scientific evidence, it remains possible that these genes represent viable *P*. *cinnamomi* RxLR effectors. It is conceivable that these effectors were expressed in avocado, but at timepoints not included in our data. More thorough expression analyses in future, including a wider range of timepoints during infection, would provide clarity as to whether these genes are upregulated at any other stages during avocado infection. Alternatively, it is possible that PcinRxLRs with relatedness to known effectors carry out their functions during infection of other host plants, and are not specifically upregulated in avocado.

## Conclusion

Hundreds of candidate *RxLR* effector genes have been predicted from genomes of *P*. *cinnamomi*, but it remains unclear how many of these are actually used by the pathogen during infection of various hosts. To our knowledge, this is the first study to identify candidate *P*. *cinnamomi RxLRs* that may play a role during the course of infection of avocado. We used transcriptome data of a susceptible avocado rootstock infected with *P*. *cinnamomi* to identify 61 putative *RxLR*s expressed during infection, which enabled the manual annotation of these genes and accurate prediction of their protein sequences. The final protein sequences of these genes were compared to known effector proteins, and putative functions could be assigned for three of the candidate RxLRs based on phylogenetic groupings and expression profiles. Twelve more candidates were shown to have diverged from characterised RxLRs in other species, though their expression patterns suggest that, should these be functional effectors, they may only carry out their proposed functions in host species other than avocado. Expression analyses suggested that nine additional effectors may have virulence roles during avocado infection, though functions could not be proposed for these candidates due to the low number of characterised RxLRs in *Phytophthora* species.

Although functional assays for *P*. *cinnamomi* RxLRs were not performed in this study due to the current inability to transform the pathogen and lack of a reliable model host plant, several promising candidate effectors were identified for future studies. The discovery of *P*. *cinnamomi RxLR*s which are specifically upregulated in avocado, or are evolutionarily related to other RxLRs, could provide a focus point for future functional studies in *P*. *cinnamomi* once reproducible transformation protocols become available for this oomycete. Furthermore, this work represents the first phylogenetic-based pipeline to identify putative functions of candidate *Phytophthora* effectors, which may help streamline future studies by pinpointing which of the hundreds of candidate RxLRs warrant further functional investigations in particular host-pathogen systems.

## Materials and methods

### Identification of candidate *RxLR*s

A bioinformatics pipeline adapted from Win *et al*. [22] was used in this study to identify candidate *RxLR* genes in the first draft genome of *P*. *cinnamomi* (*P*. *cinnamomi* var. *cinnamomi* isolate CBS 144.22), made available by JGI [48] (https://mycocosm.jgi.doe.gov/Phyci1/Phyci1.home.html). Peptides predicted by the JGI annotated genes were submitted to SignalP Version 3.0 [49] using default parameters. Peptides predicted to contain a signal peptide were searched for the presence of an RxLR motif between residues 30 and 60 of the protein sequence using perl regular expression.

Additionally, candidate *P*. *cinnamomi RxLRs* predicted by previous studies [1,23–25] were included for analysis. These included *RxLR* genes predicted by Hardham and Blackman [1] as well as Reitmann *et al*. [23] from the genome of *P*. *cinnamomi* var. *cinnamomi* isolate CBS 144.22, those predicted by McGowan and Fitzpatrick [24] from the genome of *P*. *cinnamomi* strain NZFS 3750 and *P*. *cinnamomi* strain MP94-48 [50], and those predicted by Engelbrecht *et al*. from the genome of *P*. *cinnamomi* isolate GKB4 [25]. Homologues for candidate genes predicted from other versions of the *P*. *cinnamomi* genome were found using Custom BLAST in Geneious v7.06 [51], where DNA sequences of candidate genes were searched for similarity to the DNA sequences of contigs in the most recent GKB4 genome [25]. Redundant sequences were removed, and the final list of candidate effectors was screened using SignalP 5.0 [27] to remove falsely predicted secreted proteins from the dataset.

### Plant inoculation and RNA sequencing

Expression data were obtained by dual RNA-sequencing of avocado infected with *P*. *cinnamomi*. Roots of a susceptible avocado rootstock, R0.12, were inoculated by dipping in 1.4 x 10^5^ zoospores/ml of *P*. *cinnamomi* isolate GKB4 for two hours. Plantlets were replanted in a 1:1 mixture of perlite:vermiculite, and roots were then harvested after 12 hours, 24 hours and 5 days, with three biological replicates being harvested at each timepoint. A culture of *P*. *cinnamomi* GKB4 was grown separately for 2 weeks on 20% V8 medium (200 ml clarified V8 juice, 2 g CaCO_3_, 15 g agar and distilled water to a volume of 1L) before three biological replicates of mycelia were harvested to serve as a control for this experiment.

Harvested samples were flash frozen in liquid N_2_ and stored at -70°C. They were then ground to a fine powder using an IKA® Tube Mill (IKA®, Staufen, DEU). RNA was extracted from powdered samples using a modified CTAB extraction method [52]. Extracted RNA was purified using a Qiagen RNeasy clean up kit (Qiagen, Valencia, California, USA) subsequent to treatment with DNase I (Fermentas Life Sciences, Hanover, USA). The quality and purity of extracted RNA was measured using an Agilent 2100 Bioanalyzer (Agilent Technologies, Santa Clara, CA, USA), and samples were stored at -70°C before they were sent to Novogene (Novogene Corporation Inc., Chula Vista, California, USA) for paired-end sequencing using Illumina Hiseq 2500.

### Expression analysis of candidate *RxLR*s

Trimmomatic [53] was used to trim adaptor sequences and low quality bases from RNA-Seq reads. Read quality was confirmed using FASTQC and summarised using MultiQC [54]. HISAT v2.0.6 [55] was used to align RNA-Seq reads to the *P*. *cinnamomi* GKB4 genome [25]. Transcript abundance was quantified within the RNA-Seq libraries across three time-points (12 hpi, 24 hpi, 120 hpi) using the mycelia library as a reference library–this was performed in HTSeq [56] in initial expression screens and in featureCounts [57] for subsequent expression analyses. Counts were normalised and analysed using DESeq2 [58]. Statistical significance for counts at each time point was evaluated using a confidence interval of 95%. Quantification data for candidate *RxLR* genes was extracted from DESeq2 outputs using R [59]. Heatmaps for visualisation of expression data were generated using Pheatmap v1.0.12 [60].

### Manual prediction of RxLR protein sequences

Genomic sequences for the selected *RxLR*s were manually annotated using IGV [61]. Genomic coordinates for the candidate genes in the newly assembled *P*. *cinnamomi* GKB4 genome [25] were determined using the Custom BLAST service in Geneious v7.06. Consensus sequences were generated in IGV for the selected genes based on the mapping of reads generated by the RNA-Seq experiment. Alternatively spliced transcripts were manually annotated according to the coverage of transcripts with the genomic region, and these were taken into account for manual prediction of peptide sequences.

For genes that had multiple possible loci, consensus sequences were generated for each prospective genomic location and labelled as likely repeats. Where a predicted genomic locus for a candidate *RxLR* did not have RNA-Seq reads mapping to the genomic location in question, a consensus sequence was not generated, and the region was not considered as a true genomic location for the candidate *RxLR* in this study. The remaining loci were analysed further to determine whether the candidate *RxLRs* were present in multiple copies in the genome.

Where genomic coordinates of candidate genes did not have genes already predicted for the genome by BRAKER2 [25,62] but had sufficient RNA-Seq reads mapping to the region, the genes were manually annotated using GenomeView [63]. Candidate *RxLR*s for which the BRAKER2 predicted coding regions were incorrect were also re-annotated in GenomeView using the correct genomic coordinates.

Consensus sequences for the candidate *RxLR*s were translated to their respective protein sequences in CLC Main Workbench 8.0.1, using a six-frame translation, and the correct reading frame was selected based on the presence of a signal peptide upstream of the RxLR motif within the peptide sequence. The predicted protein sequences were submitted to SignalP 5.0 to confirm that they qualified as secreted proteins. Manually curated protein sequences without signal peptides were discarded as potential effector proteins.

### Phylogenetic analysis

Candidate *P*. *cinnamomi* RxLR effectors identified in this study were aligned with previously characterised RxLR effectors in other oomycetes, which were found in a literature search for RxLRs which have been characterised in functional assays (S7 Table). Protein sequences of known effectors were obtained from Genbank (National Center for Biotechnology Information, US National Institutes of Health, USA), or those that were not available from Genbank were obtained from either the UniprotKB database (Uniprot Consortium, 2014) or supplementary information from relevant research articles. The set of peptide sequences was aligned using only the N-terminal of the proteins, similar to the method used by Goss *et al*. [64], due to the amount of variation present in C-terminal domains of these effectors. Sequences were manually edited to include only the residues up to and including the dEER motif, and when there was no dEER motif the first 80 residues were used as the N-terminal sequence for alignment. Alignment was performed using the MUSCLE alignment method [65] in Geneious v7.06. Aligned sequences were subjected to Bayesian inference analysis in MrBayes 3.2.7a [66] using the Poisson substitution model.

The proteins which formed clades with posterior probability support values above 0.5 in the phylogenetic tree were aligned to each other using the full-length amino acid sequences for each protein were used. The alignment was performed in Geneious v7.06, using a MUSCLE alignment algorithm.

### BLAST analysis

Candidate genes with expression patterns of interest were searched for similarity to other genes in the non-redundant NCBI protein database using BLASTP. The search was performed using an expect threshold of 0.00001 and output requested contained only the top hit for each candidate effector gene.

### Data deposition

Consensus sequences generated for the candidate *P*. *cinnamomi RxLR* genes named in this study have been deposited in NCBI Genbank under accession numbers MW558964-MW559024. The RNA-Seq data used in this study have been deposited in the Sequence Read Archive of NCBI Genbank under accession number PRJNA675400, with libraries named as follows: three biological replicates of mycelia were designated MS13, MS14 and MS15, three biological replicates of root samples of the susceptible rootstock at 12 hpi were named MS4, MS5 and MS6, the 24hpi library was designated MS7, MS8 and MS9 and 120hpi replicates were named MS10, MS11 and MS12.

## Supporting information

S1 Fig Initial phylogenetic analysis of non-redundant *Phytophthora cinnamomi* candidate RxLR effectors. A phylogenetic tree was produced from Bayesian inference analysis of the N-terminal regions of the *P*. *cinnamomi* candidate RxLR effectors aligned with the N-terminal regions of functionally characterised RxLRs in other oomycete species. Where groupings are supported by posterior probability (> 0.5), values are shown at nodes up to the second significant digit.
(TIF)

S2 Fig Alignment of full-length protein sequences to confirm phylogenetic inferences. For sequences which grouped within their own clades in the phylogenetic tree in Fig 1, full-length peptide sequences were aligned to confirm that similarity was not restricted to N-terminal regions. MUSCLE alignments were generated and viewed in Geneious v7.06. Alignments are shown for **a)** *Phytophthora cinnamomi* PcinRxLR52a and PcinRxLR68a with *Phytophthora infestans* PiSNE1, **b)** PcinRxLR21 and *P*. *infestans* Pi14054, **c)** PcinRxLR60 and *Phytophthora sojae* Avr3b, **d)** PcinRxLR72 and *P*. *sojae* Avh238, **e)** PcinRxLR34a, PcinRxLR35 and *Phytophthora parasitica* RxLR 2, **f)** PcinRxLR62 and *P*. *sojae* Avh146 and **g)** PcinRxLR57 and *P*. *infestans* SFI3. The high proportion of conserved residues across the different full-length alignments, and especially in (**a**), (**c**) and (**d**) above, confirmed evolutionary relatedness suggested by phylogenetic groupings in Fig 1.
(TIF)

S3 Fig Expression profiles of 28 *Phytophthora cinnamomi* candidate *RxLR*s expressed in infected avocado. Gene expression was visually represented in a heatmap based on read counts generated from dual RNA-Seq data. Expression levels are indicated as fold change of each gene normalized against expression in mycelia, and colour-coded according to the corresponding scale. Expression profiles were generated using RNA-Seq data obtained for three biological replicates of a susceptible avocado rootstock, R0.12, harvested at 12 hours-, 24 hours- and 120 hours post inoculation. Statistical significance was determined according to DESeq2 outputs, and is indicated on colour blocks for fold change relative to mycelia at relevant timepoints for each gene. *Significant expression at time-point relative to expression in mycelia at p≤0.05.
(TIF)

S1 Table Gene identities of all *Phytophthora cinnamomi RxLR* sequences used in this study. (XLSX)

S2 Table Expression data for 238 *Phytophthora cinnamomi* candidate *RxLR*s evaluated in a susceptible avocado rootstock, shown as fold change at 12 hours-, 24 hours-, and 120 hours post-inoculation, as analysed by DESeq2 from HTSeq output. (XLSX)

S3 Table Custom BLAST (blastn) results for hits to the *Phytophthora cinnamomi* GKB4 genome for 71 candidate *RxLR*s. (XLSX)

S4 Table Peptide sequences for 61 PcinRxLRs manually annotated in this study. (XLSX)

S5 Table Expression data for 61 *Phytophthora cinnamomi RxLR*s expressed in a susceptible avocado rootstock, shown as fold change at 12 hours-, 24 hours-, and 120 hours post-inoculation, as analysed by DESeq2 from featureCounts output. (XLSX)

S6 Table Results of protein BLAST searches for similarity of selected *Phytophthora cinnamomi* RxLR protein sequences to proteins in other species. (XLSX)

S7 Table List of functionally characterised RxLR effectors in oomycete species used in phylogenetic analyses. (XLSX)

S1 Appendix List of references used for characterised RxLR effectors listed in S7 Table. (DOCX)
