# Supplementary figures and images for: Expression of several Phytophthora cinnamomi putative RxLRs provides evidence for virulence roles in avocado
Source: PLoS One. 2021 Jul 14;16(7):e0254645. doi: 10.1371/journal.pone.0254645 (PMC8279351; doi:10.1371/journal.pone.0254645)

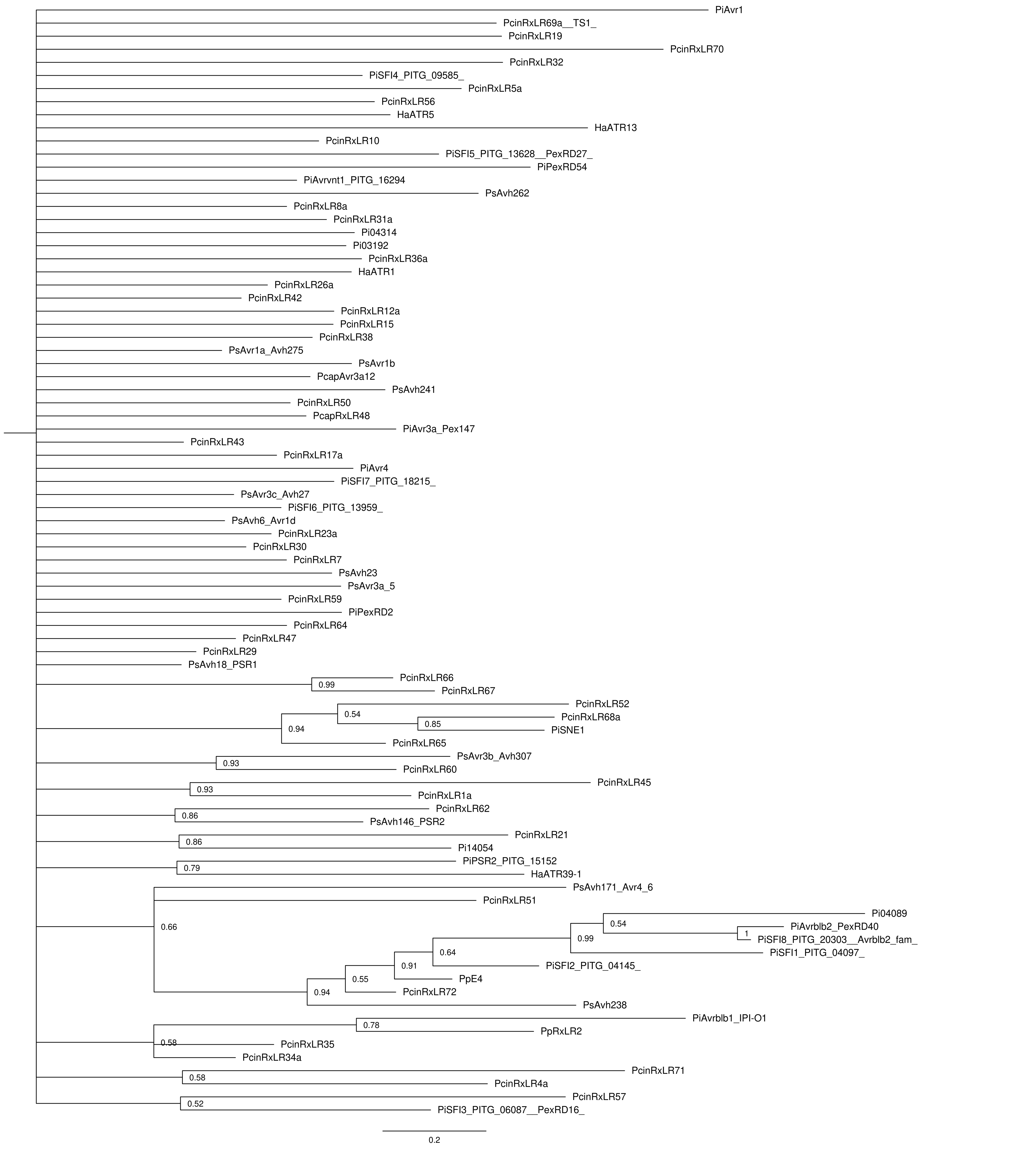

Supplement: S1 Fig — A phylogenetic tree was produced from Bayesian inference analysis of the N-terminal regions of the P. cinnamomi candidate RxLR effectors aligned with the N-terminal regions of functionally characterised RxLRs in other oomycete species. Where groupings are supported by posterior probability (> 0.5), values are shown at nodes up to the second significant digit. (TIF) [file pone.0254645.s001.tif]

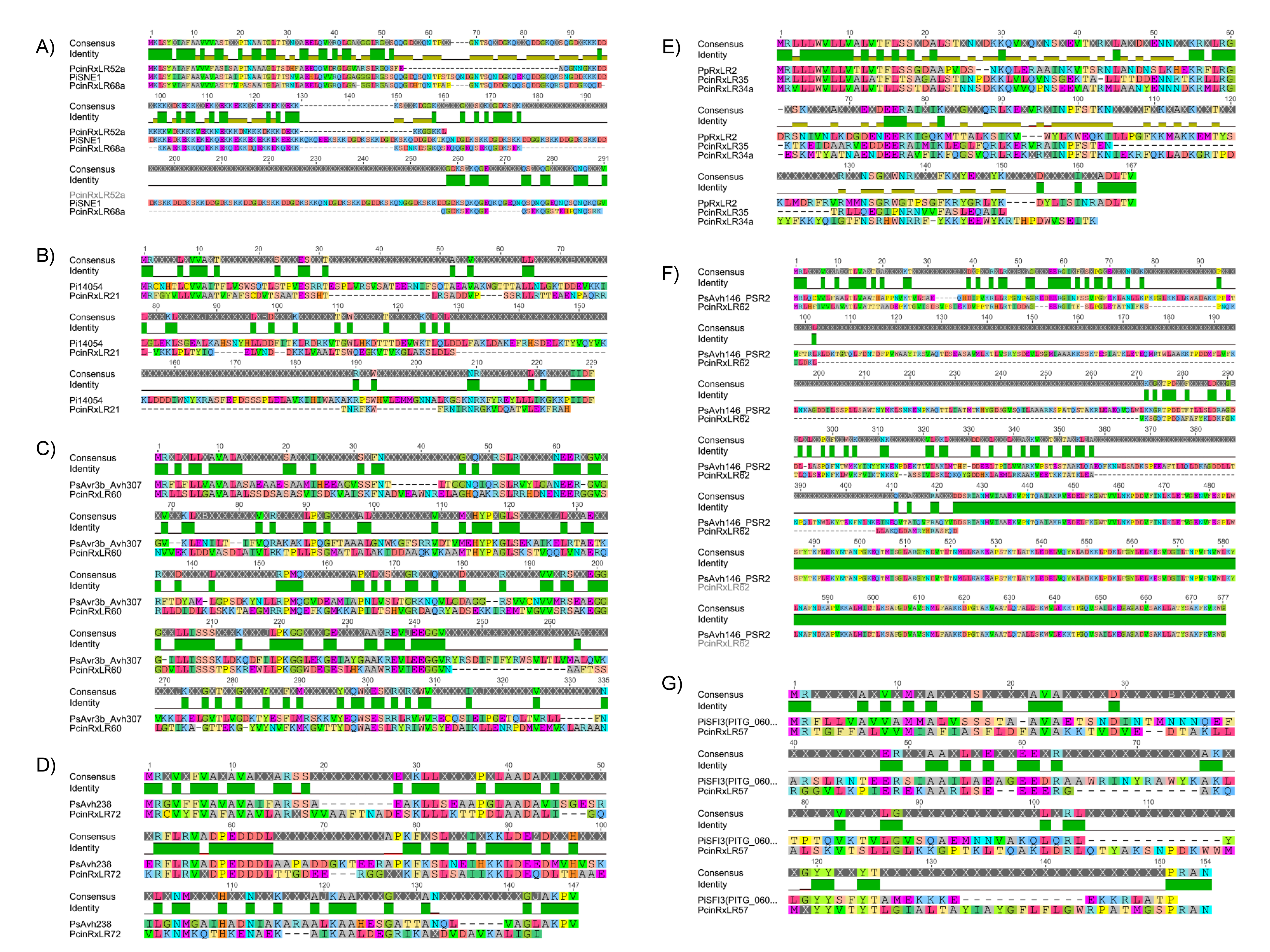

Supplement: S2 Fig — For sequences which grouped within their own clades in the phylogenetic tree in Fig 1, full-length peptide sequences were aligned to confirm that similarity was not restricted to N-terminal regions. MUSCLE alignments were generated and viewed in Geneious v7.06. Alignments are shown for a) Phytophthora cinnamomi PcinRxLR52a and PcinRxLR68a with Phytophthora infestans PiSNE1, b) PcinRxLR21 and P. infestans Pi14054, c) PcinRxLR60 and Phytophthora sojae Avr3b, d) PcinRxLR72 and P. sojae Avh238, e) PcinRxLR34a, PcinRxLR35 and Phytophthora parasitica RxLR 2, f) PcinRxLR62 and P. sojae Avh146 and g) PcinRxLR57 and P. infestans SFI3. The high proportion of conserved residues across the different full-length alignments, and especially in (a), (c) and (d) above, confirmed evolutionary relatedness suggested by phylogenetic groupings in Fig 1. (TIF) [file pone.0254645.s002.tif]

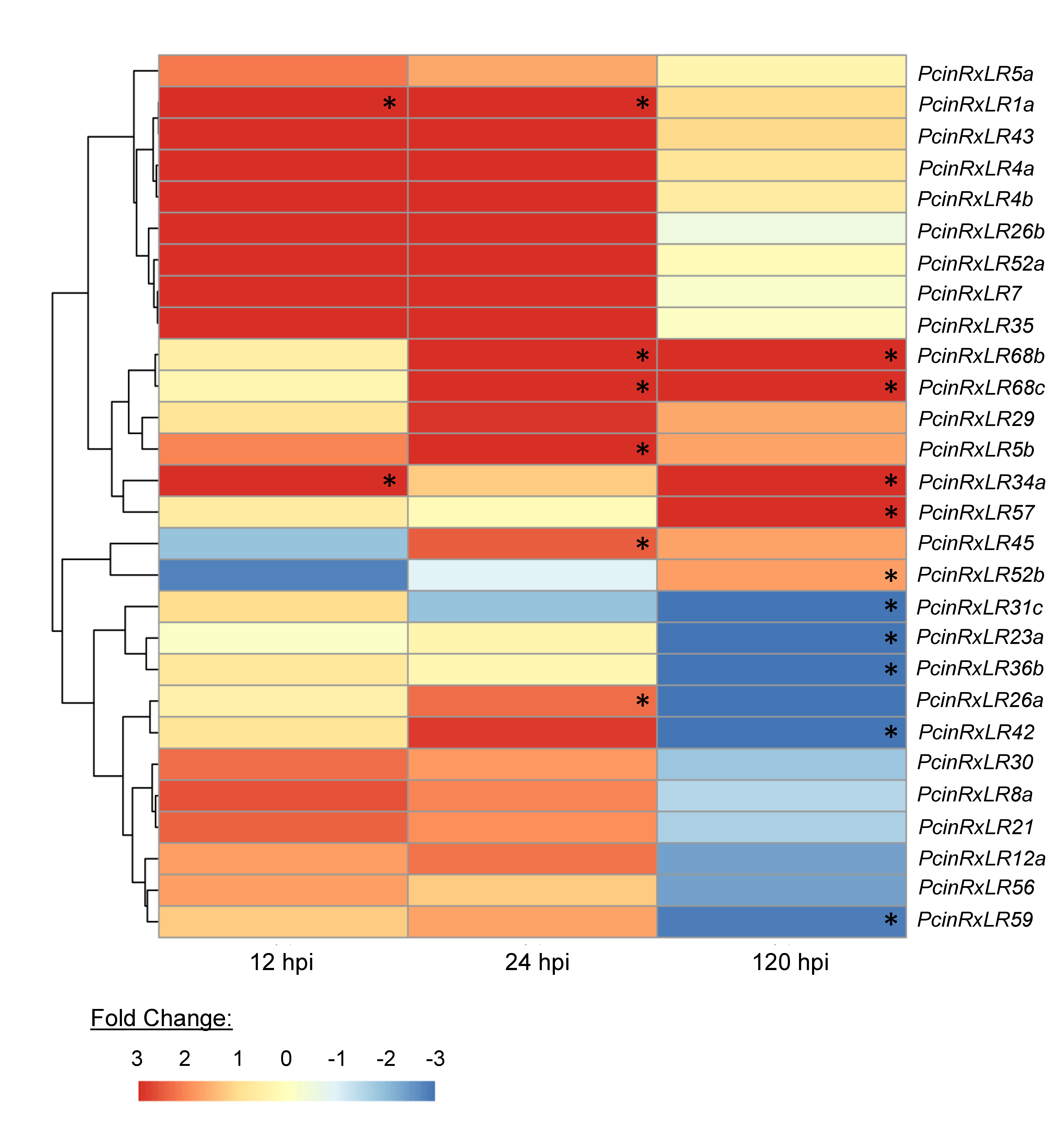

Supplement: S3 Fig — Gene expression was visually represented in a heatmap based on read counts generated from dual RNA-Seq data. Expression levels are indicated as fold change of each gene normalized against expression in mycelia, and colour-coded according to the corresponding scale. Expression profiles were generated using RNA-Seq data obtained for three biological replicates of a susceptible avocado rootstock, R0.12, harvested at 12 hours-, 24 hours- and 120 hours post inoculation. Statistical significance was determined according to DESeq2 outputs, and is indicated on colour blocks for fold change relative to mycelia at relevant timepoints for each gene. *Significant expression at time-point relative to expression in mycelia at p≤0.05. (TIF) [file pone.0254645.s003.tif]
