# Supplementary material for: Expression of several Phytophthora cinnamomi putative RxLRs provides evidence for virulence roles in avocado
Source: PLoS One. 2021 Jul 14;16(7):e0254645. doi: 10.1371/journal.pone.0254645 (PMC8279351; doi:10.1371/journal.pone.0254645)
Supplement: S1 Appendix — (DOCX) [file pone.0254645.s011.docx]

List of References for characterised RxLRs used in this study

1. Bailey K, Çevik V, Holton N, Byrne-Richardson J, Sohn KH, Coates M, et al. Molecular cloning of ATR5^Emoy2^ from *Hyaloperonospora arabidopsidis*, an avirulence determinant that triggers RPP5-mediated defense in *Arabidopsis*. Mol Plant Microbe Interact. 2011;24(7):827-38.

2. Boevink PC, Wang X, McLellan H, He Q, Naqvi S, Armstrong MR, et al. A *Phytophthora infestans* RXLR effector targets plant PP1c isoforms that promote late blight disease. Nature Communications. 2016;7:10311.

3. Bos JI, Armstrong MR, Gilroy EM, Boevink PC, Hein I, Taylor RM, et al. *Phytophthora infestans* effector AVR3a is essential for virulence and manipulates plant immunity by stabilizing host E3 ligase CMPG1. Proc Natl Acad Sci USA. 2010;107(21):9909-14.

4. Bouwmeester K, De Sain M, Weide R, Gouget A, Klamer S, Canut H, et al. The lectin receptor kinase LecRK-I. 9 is a novel *Phytophthora* resistance component and a potential host target for a RXLR effector. PLoS Pathog. 2011;7(3):e1001327.

5. Bozkurt TO, Schornack S, Win J, Shindo T, Ilyas M, Oliva R, et al. *Phytophthora infestans* effector AVRblb2 prevents secretion of a plant immune protease at the haustorial interface. Proc Natl Acad Sci USA. 2011;108(51):20832-7.

6. Chen Y, Liu Z, Halterman DA. Molecular determinants of resistance activation and suppression by *Phytophthora infestans* effector IPI-O. PLoS Pathog. 2012;8(3):e1002595.

7. Chou S, Krasileva KV, Holton JM, Steinbrenner AD, Alber T, Staskawicz BJ. *Hyaloperonospora arabidopsidis* ATR1 effector is a repeat protein with distributed recognition surfaces. Proc Natl Acad Sci USA. 2011;108(32):13323-8.

8. Dagdas YF, Belhaj K, Maqbool A, Chaparro-Garcia A, Pandey P, Petre B, et al. An effector of the Irish potato famine pathogen antagonizes a host autophagy cargo receptor. eLife. 2016;5.

9. Dalio R, Maximo H, Oliveira T, Dias R, Breton M, Felizatti H, et al. *Phytophthora parasitica* effector PpRxLR2 suppresses *Nicotiana benthamiana* immunity. Mol Plant Microbe Interact. 2018;31(4):481-93.

10. de Vries S, von Dahlen JK, Uhlmann C, Schnake A, Kloesges T, Rose LE. Signatures of selection and host‐adapted gene expression of the *Phytophthora infestans* RNA silencing suppressor PSR2. Mol Plant Pathol. 2017;18(1):110-24.

11. Dong S, Yin W, Kong G, Yang X, Qutob D, Chen Q, et al. *Phytophthora sojae* avirulence effector Avr3b is a secreted NADH and ADP-ribose pyrophosphorylase that modulates plant immunity. PLoS Pathog. 2011b;7(11):e1002353.

12. Dong S, Yu D, Cui L, Qutob D, Tedman-Jones J, Kale SD, et al. Sequence variants of the *Phytophthora sojae* RXLR effector Avr3a/5 are differentially recognized by Rps3a and Rps5 in soybean. PLoS One. 2011a;6(7):1-8.

13. Dou D, Kale SD, Liu T, Tang Q, Wang X, Arredondo FD, et al. Different domains of *Phytophthora sojae* effector Avr4/6 are recognized by soybean resistance genes *Rps 4* and *Rps 6*. Mol Plant Microbe Interact. 2010;23(4):425-35.

14. Dou D, Kale SD, Wang X, Chen Y, Wang Q, Wang X, et al. Conserved C-terminal motifs required for avirulence and suppression of cell death by *Phytophthora sojae* effector Avr1b. Plant Cell. 2008b;20(4):1118-33.

15. Dou D, Kale SD, Wang X, Jiang RH, Bruce NA, Arredondo FD, et al. RXLR-mediated entry of *Phytophthora sojae* effector Avr1b into soybean cells does not require pathogen-encoded machinery. Plant Cell. 2008a;20(7):1930-47.

16. Du Y, Mpina MH, Birch PR, Bouwmeester K, Govers F. *Phytophthora infestans* RXLR effector AVR1 interacts with exocyst component Sec5 to manipulate plant immunity. Plant Physiology. 2015;169(3):1975-90.

17. Fang Y, Tyler BM. Efficient disruption and replacement of an effector gene in the oomycete *Phytophthora sojae* using CRISPR/Cas9. Mol Plant Pathol. 2016;17(1):127-39.

18. Gilroy EM, Taylor RM, Hein I, Boevink P, Sadanandom A, Birch PR. CMPG1‐dependent cell death follows perception of diverse pathogen elicitors at the host plasma membrane and is suppressed by *Phytophthora infestans* RXLR effector AVR3a. New Phytologist. 2011b;190(3):653-66.

19. Goritschnig S, Krasileva KV, Dahlbeck D, Staskawicz BJ. Computational prediction and molecular characterization of an oomycete effector and the cognate Arabidopsis resistance gene. PLOS genetics. 2012;8(2):e1002502.

20. He Q, McLellan H, Hughes RK, Boevink PC, Armstrong M, Lu Y, et al. *Phytophthora infestans* effector SFI 3 targets potato UBK to suppress early immune transcriptional responses. New Phytologist. 2019;222(1):438-54.

21. Huang G, Liu Z, Gu B, Zhao H, Jia J, Fan G, et al. An RXLR effector secreted by *Phytophthora parasitica* is a virulence factor and triggers cell death in various plants. Mol Plant Pathol. 2019;20(3):356-71.

22. Jing M, Guo B, Li H, Yang B, Wang H, Kong G, et al. A *Phytophthora sojae* effector suppresses endoplasmic reticulum stress-mediated immunity by stabilizing plant Binding immunoglobulin Proteins. Nature Communications. 2016;7:11685.

23. Kelley BS, Lee SJ, Damasceno CM, Chakravarthy S, Kim BD, Martin GB, et al. A secreted effector protein (SNE1) from *Phytophthora infestans* is a broadly acting suppressor of programmed cell death. Plant J. 2010;62(3):357-66.

24. King SR, McLellan H, Boevink PC, Armstrong MR, Bukharova T, Sukarta O, et al. *Phytophthora infestans* RXLR effector PexRD2 interacts with host MAPKKKε to suppress plant immune signaling. The Plant Cell. 2014;26:1345-59.

25. Kong L, Qiu X, Kang J, Wang Y, Chen H, Huang J, et al. A *Phytophthora* Effector Manipulates Host Histone Acetylation and Reprograms Defense Gene Expression to Promote Infection. Current Biology. 2017;27(7):981-91.

26. Leonelli L, Pelton J, Schoeffler A, Dahlbeck D, Berger J, Wemmer DE, et al. Structural elucidation and functional characterization of the *Hyaloperonospora arabidopsidis* effector protein ATR13. PLoS Pathog. 2011;7(12):e1002428.

27. Li Q, Chen Y, Wang J, Zou F, Jia Y, Shen D, et al. A *Phytophthora capsici* virulence effector associates with NPR1 and suppresses plant immune responses. Phytopathol Res. 2019a;1(6).

28. McLellan H, Boevink PC, Armstrong MR, Pritchard L, Gomez S, Morales J, et al. An RxLR effector from *Phytophthora infestans* prevents re-localisation of two plant NAC transcription factors from the endoplasmic reticulum to the nucleus. PLoS Pathog. 2013;9(10):e1003670.

29. Na R, Yu D, Chapman BP, Zhang Y, Kuflu K, Austin R, et al. Genome re-sequencing and functional analysis places the *Phytophthora sojae* avirulence genes *Avr1c* and *Avr1a* in a tandem repeat at a single locus. PLoS One. 2014;9(2):e89738.

30. Na R, Yu D, Qutob D, Zhao J, Gijzen M. Deletion of the *Phytophthora sojae* avirulence gene *Avr1d* causes gain of virulence on *Rps*1d. Mol Plant Microbe Interact. 2013;26(8):969-76.

31. Qiao Y, Liu L, Xiong Q, Flores C, Wong J, Shi J, et al. Oomycete pathogens encode RNA silencing suppressors. Nat Genet. 2013;45(3):330-3.

32. Qiao Y, Shi J, Zhai Y, Hou Y, Ma W. *Phytophthora* effector targets a novel component of small RNA pathway in plants to promote infection. Proc Natl Acad Sci USA. 2015;112(18):5850-5.

33. Qutob D, Tedman-Jones J, Dong S, Kuflu K, Pham H, Wang Y, et al. Copy number variation and transcriptional polymorphisms of *Phytophthora sojae* RXLR effector genes *Avr1a* and *Avr3a*. PLoS One. 2009;4(4):e5066.

34. Senchou V, Weide R, Carrasco A, Bouyssou H, Pont-Lezica R, Govers F, et al. High affinity recognition of a *Phytophthora* protein by *Arabidopsis* via an RGD motif. Cellular and Molecular Life Sciences. 2004;61(4):502-9.

35. Stefańczyk E, Brylińska M, Brurberg M, Naerstad R, Elameen A, Sobkowiak S, et al. Diversity of *Avr‐vnt1* and *AvrSmira1* effector genes in Polish and Norwegian populations of *Phytophthora infestans*. Plant Pathol. 2018;67(8):1792-802.

36. van Poppel PM, Guo J, van de Vondervoort PJ, Jung MW, Birch PR, Whisson SC, et al. The *Phytophthora infestan*s avirulence gene *Avr4* encodes an RXLR-dEER effector. Mol Plant Microbe Interact. 2008;21(11):1460-70.

37. Vetukuri RR, Kushwaha S, Sen D, Whisson SC, Lamour K, Grenville-Briggs L. Genome sequence resource for the oomycete taro pathogen *Phytophthora colocasiae*. Mol Plant Microbe Interact. 2018.

38. Wang Q, Han C, Ferreira AO, Yu X, Ye W, Tripathy S, et al. Transcriptional programming and functional interactions within the *Phytophthora sojae* RXLR effector repertoire. Plant Cell. 2011;23(6):2064-86.

39. Xiong Q, Ye W, Choi D, Wong J, Qiao Y, Tao K, et al. *Phytophthora* suppressor of RNA silencing 2 is a conserved RxLR effector that promotes infection in soybean and *Arabidopsis thaliana*. Mol Plant Microbe Interact. 2014;27(12):1379-89.

40. Yang B, Wang Y, Guo B, Jing M, Zhou H, Li Y, et al. The *Phytophthora sojae* RXLR effector Avh238 destabilizes soybean Type2 GmACSs to suppress ethylene biosynthesis and promote infection. New Phytologist. 2019;222(1):425-37.

41. Yin W, Dong S, Zhai L, Lin Y, Zheng X, Wang Y. The *Phytophthora sojae Avr1d* gene encodes an RxLR-dEER effector with presence and absence polymorphisms among pathogen strains. Mol Plant Microbe Interact. 2013;26(8):958-68.

42. Zheng X, McLellan H, Fraiture M, Liu X, Boevink PC, Gilroy EM, et al. Functionally redundant RXLR effectors from *Phytophthora infestans* act at different steps to suppress early flg22-triggered immunity. PLoS Pathog. 2014;10(4):e1004057.

43. Zheng X, Wagener N, McLellan H, Boevink PC, Hua C, Birch PR, et al. *Phytophthora infestans* RXLR effector SFI 5 requires association with calmodulin for PTI/MTI suppressing activity. New Phytologist. 2018;219(4):1433-46.
